# Monocytes Elicit a Neutrophil-Independent Th1/Th17 Response Upon Immunization With a Mincle-Dependent Glycolipid Adjuvant

**DOI:** 10.3389/fimmu.2022.880474

**Published:** 2022-05-02

**Authors:** Christiane Desel, Peter J. Murray, Christian H. K. Lehmann, Lukas Heger, Dennis Christensen, Peter Andersen, Matthias Mack, Diana Dudziak, Roland Lang

**Affiliations:** ^1^ Institute of Clinical Microbiology, Immunology and Hygiene, University Hospital Erlangen, Friedrich-Alexander-Universität Erlangen-Nürnberg, Erlangen, Germany; ^2^ Department of Infectious Disease, St. Jude Children’s Research Hospital, Memphis, TN, United States; ^3^ Department of Immunology, St. Jude Children’s Research Hospital, Memphis, TN, United States; ^4^ Department of Dermatology, Laboratory of Dendritic Cell Biology, University Hospital Erlangen, Friedrich-Alexander-Universität Erlangen-Nürnberg, Erlangen, Germany; ^5^ Department of Infectious Disease Immunology, Statens Serum Institute, Copenhagen, Denmark; ^6^ Department of Nephrology, University Hospital Regensburg, Regensburg, Germany

**Keywords:** adjuvant, vaccination, TDB, Th17, monocytes, neutrophils

## Abstract

Successful subunit vaccination with recombinant proteins requires adjuvants. The glycolipid trehalose-dibehenate (TDB), a synthetic analog of the mycobacterial cord factor, potently induces Th1 and Th17 immune responses and is a candidate adjuvant for human immunization. TDB binds to the C-type lectin receptor Mincle and triggers Syk-Card9-dependent APC activation. In addition, interleukin (IL)-1 receptor/MyD88-dependent signaling is required for TDB adjuvanticity. The role of different innate immune cell types in adjuvant-stimulated Th1/Th17 responses is not well characterized. We investigated cell recruitment to the site of injection (SOI) and to the draining lymph nodes (dLNs) after immunization with the TDB containing adjuvant CAF01 in a protein-based vaccine. Recruitment of monocytes and neutrophils to the SOI and the dramatic increase in lymph node cellularity was partially dependent on both Mincle and MyD88. Despite their large numbers at the SOI, neutrophils were dispensable for the induction of Th1/Th17 responses. In contrast, CCR2-dependent monocyte recruitment was essential for the induction of Th1/Th17 cells. Transport of adjuvant to the dLN did not require Mincle, MyD88, or CCR2. Together, adjuvanticity conferred by monocytes can be separated at the cellular level from potential tissue damage by neutrophils.

## Introduction

The co-administration of adjuvants is required to induce T-cell immunity to protein antigens. Unfortunately, the lack of understanding the molecular and cellular mechanisms of adjuvant-induced immune responses hinders rational vaccine design and deployment, especially for emerging infectious diseases, or vaccines where antigen sparing (e.g., influenza) is an objective. Adjuvants in vaccines licensed for humans include aluminum salts, oil-in-water emulsions (MF59, AS03), liposomes (AS01), CpG oligonucleotides, and virosomes. While many of these induce strong antibody responses, most of them only weakly provoke Th1 and especially Th17 immunity, which is crucial for defense against intracellular pathogens, e.g., *Mycobacterium tuberculosis* (1). The use of molecularly defined adjuvants is a key component required for successful development of novel recombinant subunit vaccines. However, despite recent progress (2), to date, such adjuvants for safe and efficient use in humans are still lacking (3).

DDA/TDB [also known as CAF01 (4)] is a next generation synthetic adjuvant. DDA/TDB has been successfully evaluated in phase I clinical studies for vaccination with, e.g., the recombinant *M. tuberculosis* fusion protein Ag85B-ESAT-6 (H1) (5) and with the recombinant chlamydia protein CTH522 (6). DDA/TDB is a mixture of cationic dimethyldioctadecylammonium (DDA) surfactant lipid-based liposomes containing trehalose-6,6-dibehenate (TDB), the synthetic analog of the mycobacterial cord factor trehalose-6,6-dimycolate (TDM). In contrast to, e.g., aluminum salts and the TLR9-triggering adjuvant CpG, it was shown that DDA/TDB induces strong and long-lasting Th17 memory responses in mice (7–11). TDB and TDM bind to the C-type lectin receptor Mincle (Clec4e) and activate antigen-presenting cells (APC) *via* the FcRγ-Syk-Card9 pathway (10, 12, 13). Consequently, deletion of the Mincle receptor or the FcRγ-Card9 pathway in mice strongly reduces the Th17 adjuvant activity of TDB (13, 14) and TDM (15). In addition, MyD88-dependent signaling *via* IL-1R is required for induction of DDA/TDB-mediated generation of vaccine-induced immunity (14). Expression of Mincle is strongly regulated by cytokines, with IL-4 suppressing Mincle messenger RNA (mRNA) and protein levels in mouse and human macrophages (16, 17), whereas tumor necrosis factor (TNF) is essential for the upregulation of Mincle and Th17 adjuvanticity by DDA/TDB (18). In addition to the cord factor analog TDB, other trehalose esters have been explored as Mincle agonists in the search for novel Th17-inducing adjuvants (19–25).

Beyond the definition of innate immune receptors and pathways essential for adjuvant activity, the question of which APC are required for the generation of T-cell responses after immunization is pivotal for rational vaccine design. Current thinking suggests that vaccine immunogenicity depends on direct dendritic cell (DC) targeting and activating capacity of the administered adjuvant. However, following injection of fluorescently labeled DDA/TDB, only a minute fraction of CD11c^+^ lymph node DC contained antigen/adjuvant and became activated (26). Therefore, whether this exquisite targeting of DC in the lymph node is the major prerequisite for a successful adjuvant remains unclear. Many adjuvant formulations, including the classical human adjuvant aluminum hydroxide cause strong recruitment of innate immune cells to the SOI (27, 28). Surprisingly, aluminum hydroxide has no stimulatory effect on DC *in vitro* (29). Instead, *in vivo* experiments revealed that, in the presence of aluminum hydroxide, antigen was taken up by recruited inflammatory monocytes and transported to the dLN. These cells differentiate into inflammatory DC and contribute to adaptive immune responses (29). Subcutaneous immunization with DDA/TDB liposomes in mice causes robust and sustained local swelling and cellular infiltration (10, 13, 14). However, the detailed cellular composition and contribution of the local inflammatory response to successful adjuvanticity are unknown. Here, we investigated the recruitment of APC to the SOI and its dependence on the Mincle receptor and MyD88-signaling and identified CCR2^+^ monocytes, but not neutrophils, as critical for the induction of Th1/Th17 responses by the DDA/TDB adjuvant *in vivo*.

## Results

### The Vaccine Adjuvant DDA/TDB Induces Rapid Influx of Neutrophils and Monocytes

**Figure 1:**
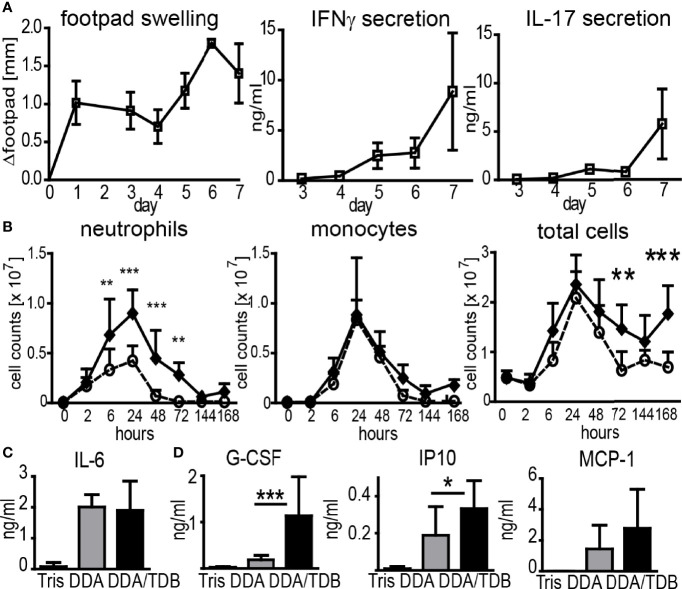
DDA/TDB induces rapid influx of neutrophils and monocytes to the SOI. **(A)** Kinetics of footpad swelling and IFNγ and IL-17 secretion after s.c. injection of DDA/TDB/H1. Re-stimulation of pooled cells from popliteal and inguinal lymph nodes. **(B)** Recruitment kinetics after i.p. injection of DDA (open circles) or DDA/TDB (closed spheres). For gating strategies, see **Supplementary Figure S1**. Pooled data from eight independent experiments, total n = 5–9 mice per group/time point. **(C)** IL-6 2 h and **(D)** G-CSF, IP10, and MCP-1 24 h p.i. in peritoneal lavage; Tris (vehicle control), DDA, or DDA/TDB. Pooled data from four independent experiments, a total of 6–12 mice per group. Significance tested by two-way ANOVA and Bonferroni correction **(B)** or t-test **(C, D)**. *p < 0.05, **p < 0.01, ***p < 0.001.

Subcutaneous immunization with recombinant protein delivered in the adjuvant formulation DDA/TDB induced inflammation at the SOI in the footpad, peaking at days 6–7 and coinciding with the appearance of antigen-specific Th1/Th17 cells in the dLN (**Figure 1A**). We have previously demonstrated that interferon gamma (IFNγ) and interleukin (IL)-17 in this setting is primarily secreted by antigen-specific CD4^+^ T cells (14). To dissect the cellular mechanisms of the DDA/TDB adjuvant effect, we determined recruitment kinetics of innate and adaptive immune cells to the SOI. For ease of cell isolation, we first performed intra-peritoneal (i.p.) injections and harvested cells by peritoneal lavage. Description and examples of gating strategies for flow cytometry are provided in **Supplementary Figure S1A**. We found that the cellular influx after injection of DDA/TDB or DDA liposomes peaked after 24 h and was initially composed almost entirely of neutrophils and monocytes, with neutrophils arriving slightly earlier (**Figure 1B**). Significantly higher numbers of neutrophils were the only TDB-specific effect observed in the first 3 days, whereas monocytes and all other cell populations were recruited equally after injection of DDA liposomes (**Figure 1B**; **Supplementary Figure S1B**). NK, B, γδTCR^+^, CD4^+^, and CD8^+^ T cells and DC were detected at later time points and in significantly higher numbers in the DDA/TDB group (**Supplementary Figure S1B**). Higher numbers of recruited neutrophils at the peak of the inflammatory response to DDA/TDB coincided with enhanced release of granulocyte-colony stimulating factor (G-CSF) and interferon-inducible protein 10 (IP10), whereas IL-6 and monocyte chemoattractant protein-1 (MCP-1) secretion was comparable between DDA and DDA/TDB (**Figures 1C, D**). Neutrophils and monocytes were also the main cell populations recruited to the SOI upon s.c. footpad immunization, albeit with differing kinetics (**Supplementary Figure S1C**).

### Mincle and MyD88 Contribute to Monocyte/Neutrophil Recruitment and Increased Lymph Node Cellularity

**Figure 2:**
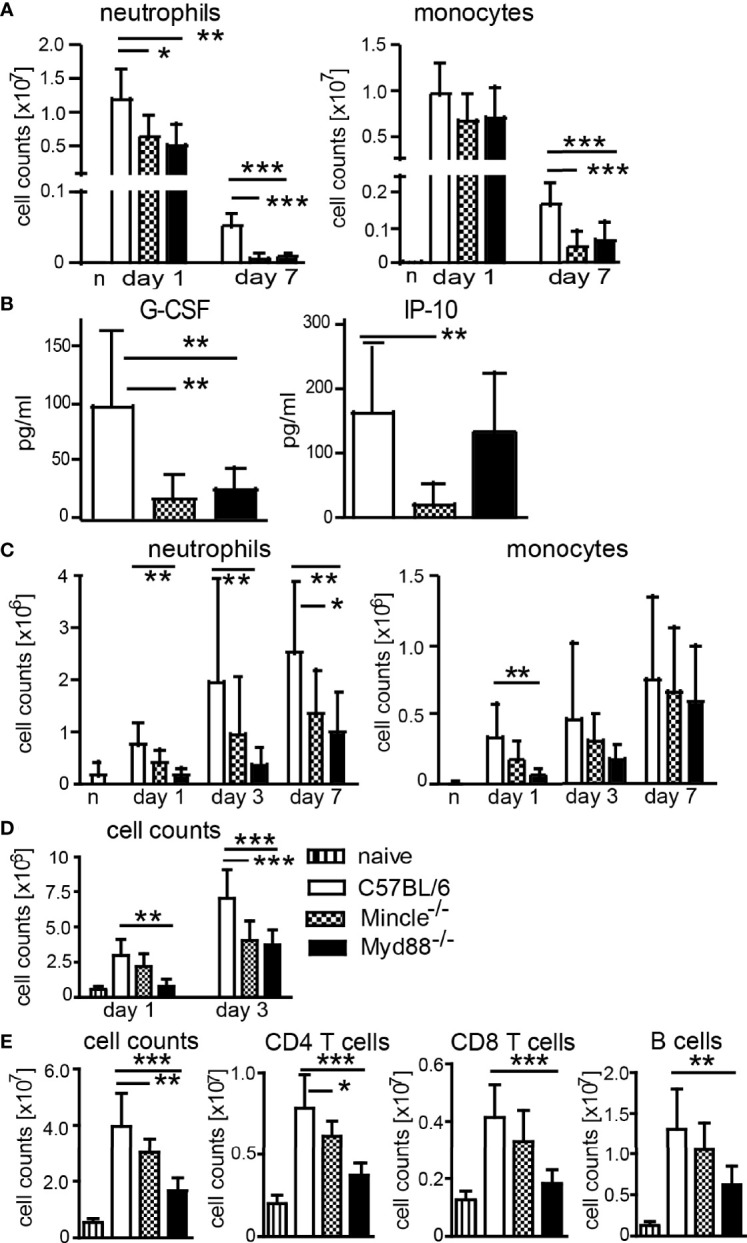
Mincle- and MyD88-dependent pathways contribute to local monocyte and neutrophil recruitment and increased lymph node cellularity. **(A)** Cell numbers at d1 and d7 and **(B)** chemokines in peritonal lavage 7 days post-DDA/TDB i.p. Pooled data from five independent experiments with a total of n=6–10 mice per group and time point. **(C)** Cellular influx after NBD-DDA/TDB s.c. footpad immunization. Pooled data from seven independent experiments with a total of n=6–9 mice per group/time point. n, naive C57BL/6 control. **(D)** Numbers of cells isolated from popliteal lymph nodes 1 and 3 days p.i., s.c. footpad immunization with NBD-DDA/TDB. Pooled data from two independent experiments with a total of five to six mice per group/time point. **(E)** Cell numbers in pooled popliteal and inguinal lymph nodes 7 days p.i. All data presented as mean+SD. Significance tested by one-way ANOVA for each time point and Dunnett’s post-test with immunized C57BL/6 as control group. *p < 0.05, **p < 0.01, ***p < 0.001. For gating, see **Supplementary Figure S1**.

Generation of IFNγ- and IL-17-producing T cells after immunization with DDA/TDB as adjuvant was shown to require the C-type lectin receptor Mincle and the TLR/IL-1R adaptor MyD88 (13, 14). We therefore asked whether cell recruitment after immunization is dependent on Mincle and MyD88. We performed flow cytometry analyses of cells isolated from the peritoneum in *Mincle* ^−/−^ or *Myd88* ^−/−^ mice upon DDA/TDB injection and found that the TDB-mediated neutrophil influx was dependent on Mincle and MyD88 signaling, whereas early recruitment of monocytes was not significantly altered by knocking out these pathways (**Figure 2A**). However, sustained cellular influx at d7 was only observed with DDA/TDB (**Figure 1B**) and required Mincle and MyD88 (**Figure 2A**). In line with this, pro-inflammatory mediators were reduced in the absence of Mincle or MyD88 at the later phase of the inflammatory response (**Figure 2B**). Similar observations of dependence on Mincle and MyD88 were made after s.c. footpad immunization, although the overall kinetics (peak 24 h lavage vs. 6–7 days footpad) differed between the routes of immunization (**Figure 2C**). Furthermore, the size of the draining popliteal lymph node increased rapidly after injection of DDA/TDB, with an approximately 5-fold increase in total cell number during the first 24 h and more than 10-fold after 3 days p.i. (**Figure 2D**). The initial increase was Mincle independent but required MyD88, whereas 3 days p.i. cell numbers were significantly reduced in the absence of both Mincle and MyD88 (**Figure 2D**). Seven days p.i., when antigen-specific CD4^+^ T cells can be reliably detected, we found substantially increased cell numbers in popliteal and inguinal lymph nodes; this was again dependent on Mincle and MyD88, with a similar reduction in CD4^+^ and CD8^+^ T cells and B cells (**Figure 2E**).

### Acquisition of Adjuvant by dLN APC

Even though neutrophils and monocytes were the main cell populations recruited to the SOI (**Supplementary Figure S1C**), their numbers in the dLN were quite low and only reduced in *Myd88* ^−/−^ mice at 24 h p.i. (**Supplementary Figure S2A**). Numbers of DC did not significantly differ between the immunized groups, whereas the increase in macrophages required MyD88 (**Supplementary Figure S2A**). To visualize the cellular uptake and fate of the vaccine, we injected 7-nitrobenzo-2-oxa-1,3-diazole (NBD) fluorescently labeled DDA/TDB in the footpad and analyzed the cells in the dLN by flow cytometry (**Supplementary Figure S2B**). Our data revealed no differences in the numbers of NBD-DDA/TDB-positive total cells, neutrophils, monocytes, or DC in wild type, *Mincle* ^−/−^, and *Myd88* ^−/−^ mice (**Supplementary Figure S2B**). Thus, we conclude that the adjuvant was detected in a small proportion of APC in the dLN independent of Mincle or MyD88, arguing against differences in transport as an explanation for the Mincle and MyD88 dependence of adjuvant activity.

### Depletion of Monocytes, but Not of Neutrophils, Abrogates Adjuvant Effect of DDA/TDB

**Figure 3:**
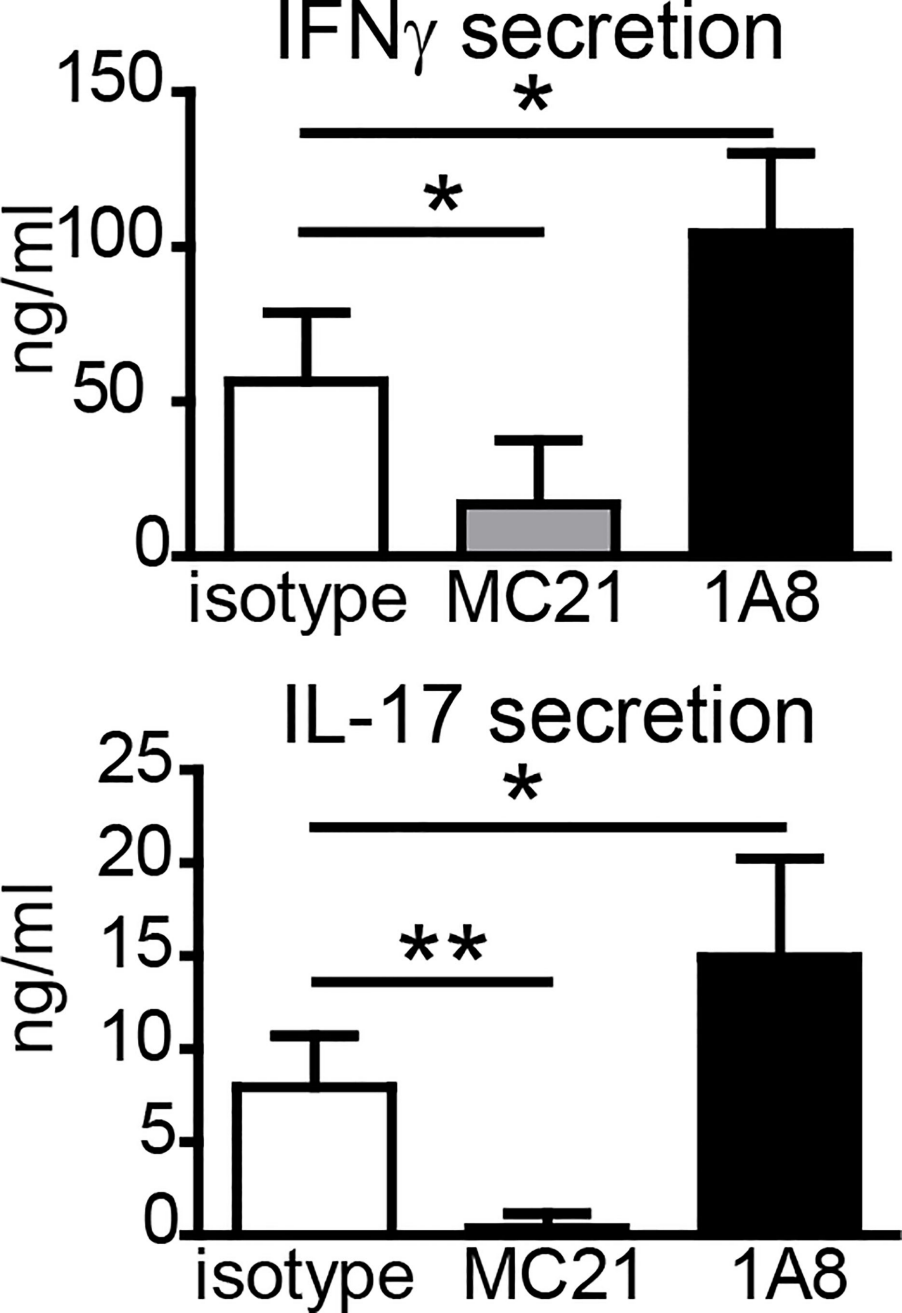
Effect of antibody-mediated depletion of monocytes or neutrophils on adjuvanticity of DDA/TDB. Antigen-specific IFNγ and IL-17 secretion of pooled cells from popliteal and inguinal lymph nodes 7 days p.i.; DDA/TDB/H1 s.c. footpad. Depletion with monoclonal antibodies MC-21 (monocytes) or 1A8 (neutrophils); isotype control (rat IgG2_b_κ). Mean+SD of 1 of the three representative experiments with five mice/group. Significance tested by one-way ANOVA and Dunnett’s post-test with isotype as control group; *p < 0.05 and **p < 0.01.

Since neutrophils and monocytes were the main cell types at the SOI, we asked whether they were essential for the generation of antigen-specific T-cell responses upon vaccination. Many studies have used an anti-Gr-1 antibody (clone RB6-8C5) to deplete neutrophils. However, this clone recognizes not only the neutrophil-specific Ly6G but also Ly6C, which is more widely expressed, including monocytes, several DC subsets, and activated T cells (30, 31). Thus, we used the Ly6G-specific antibody 1A8 for specific depletion of neutrophils (31). CCR2 is expressed highly on inflammatory Ly6C^hi^ monocytes, and we employed the CCR2-specific antibody MC-21 to selectively deplete monocytes (32–34). Despite efficient reduction in neutrophils (**Supplementary Figures S3A, B**), the Ly6G-specific antibody 1A8 did not impair Th1/Th17 cell generation (**Figure 3**); in two out of three independent experiments, IFNγ and IL-17 secretion was even increased if neutrophils were depleted. Depletion of monocytes with MC-21 can only be achieved for up to 5 days (33); thus, we observed strongly reduced numbers of monocytes in blood, footpad, and lymph node 24 h but not 7 days p.i. (**Supplementary Figures S3B, C**). Nevertheless, depletion of monocytes with the CCR2-specific MC-21 antibody resulted in a strong reduction in antigen-specific Th1/Th17 T cell generation (**Figure 3**).

### Genetically Deficient Mice Confirm Essential Role of Monocytes but not Neutrophils

**Figure 4:**
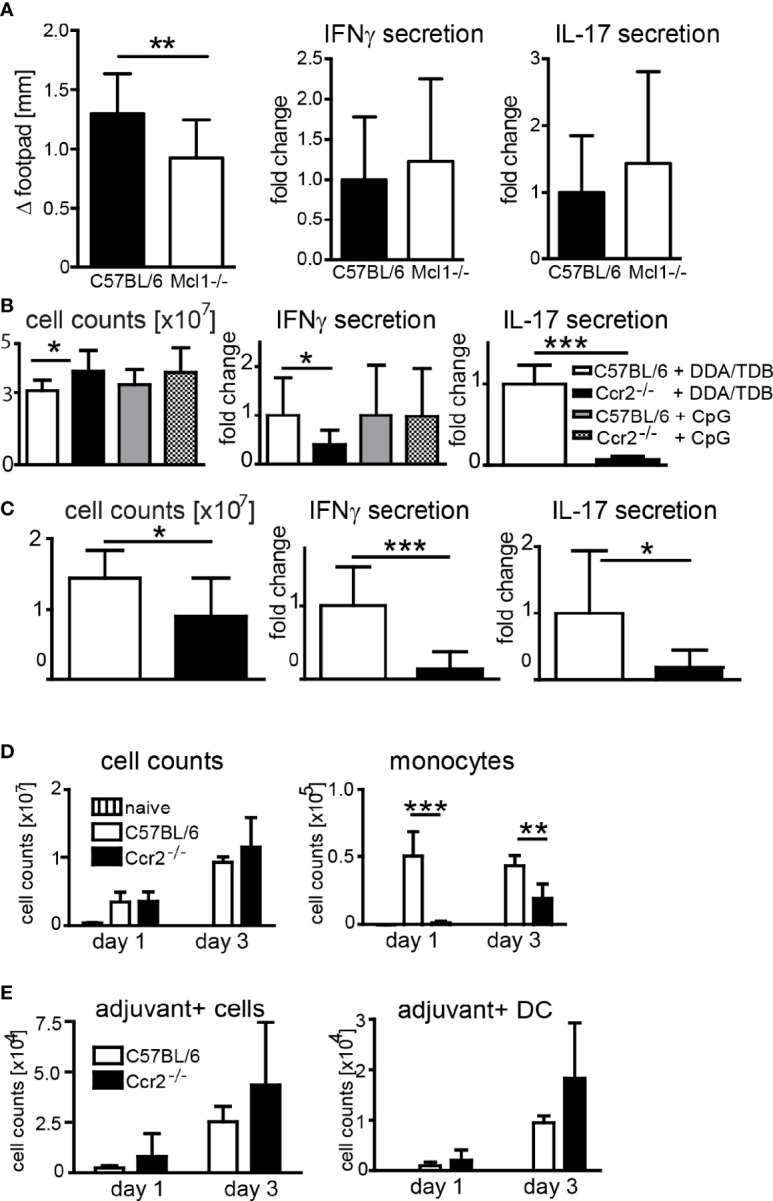
Effect of genetic depletion of neutrophils or CCR2 on adjuvanticity of DDA/TDB. **(A)** Footpad swelling and IFNγ and IL-17 secretion from popliteal lymph nodes of *Mcl1*-deficient and *Mcl1* ^+/+^; LysM-Cre^+^ control mice 7 days after DDA/TDB/H1 s.c. footpad immunization. N=7/9, pooled from two independent experiments. **(B, C)** Cell counts, IFNγ, and IL-17 secretion from pooled popliteal and inguinal lymph nodes of C57BL/6 or *Ccr2* ^−/−^ mice **(B)** 7 days post footpad or **(C)** 28 days post s.c. base of tail immunization (2´ 14-day interval). Pooled data from two independent experiments each with a total of n=10 mice per group. Cytokine secretion normalized to immunized control mice; all data presented as mean+SD. Significance tested by t-test; *p < 0.05 and ***p < 0.001. **(D)** Numbers of total cells and monocytes and **(E)** of adjuvant^+^ total cells and DC, in popliteal lymph nodes 1 and 3 days p.i., s.c. footpad immunization with NBD-DDA/TDB. Pooled data from two independent experiments with a total of five to six mice per group/time point. All data presented as mean+SD. Significance tested by one-way ANOVA and Dunnett’s post-test with immunized C57BL/6 as control group; *p < 0.05, **p < 0.01, and ***p < 0.001. For gating, see **Supplementary Figure S1**.

In addition to antibody-mediated depletion, we used transgenic mouse models to determine the contribution of neutrophils and monocytes to the adjuvant effect of DDA/TDB. Conditional Mcl-1 knockout mice deleting the gene in myeloid cells lack granulocytes but have normal numbers of monocytes (35). When we immunized *Mcl1* ^flox/flox^; LysM-Cre^+^ mice, we found slightly reduced footpad swelling, but comparable IFNγ and IL-17 responses as in control mice (**Figure 4A**), corroborating that neutrophils are dispensable for Th1/Th17 adjuvanticity. Emigration of Ly6C^hi^ monocytes from the bone marrow requires CCR2, and *Ccr2* ^−/−^ mice therefore lack inflammatory monocytes in the peripheral blood (36). To further test the importance of monocyte recruitment for the adjuvant effect of DDA/TDB, we therefore immunized *Ccr2* ^−/−^ mice. The generation of antigen-specific Th1, and even more so Th17, responses was severely reduced in *Ccr2* ^−/−^ mice (**Figure 4B**). Prime-boost immunizations confirmed the non-redundant role of CCR2 for generation of antigen-specific Th1/Th17 cells (**Figure 4C**). We found that T cells from *Ccr2* ^−/−^ mice did not have a general defect in IL-17/IFNγ production, since they secreted comparable amounts of these cytokines after polyclonal *in vitro* stimulation (**Supplementary Figure S4A**). Furthermore, *Ccr2* ^−/−^ bone marrow-derived macrophages and DC were equally responsive to glycolipid stimulation (**Supplementary Figure S4B**), arguing against differences in general APC activation as an explanation for the CCR2 dependence of DDA/TDB adjuvanticity. We also used the TLR9 ligand CpG ODN 1826 as adjuvant for immunization with H1, since this soluble adjuvant freely drains to the lymphatic system. Immunization with CpG did not cause footpad swelling or local cell recruitment (**Supplementary Figure S4C**) and fails to trigger a Th17 response (10). However, the Th1 response induced by CpG ODN 1826 was not significantly reduced in the absence of CCR2 (**Figure 4B**). Together, the results from antibody-mediated depletion and knockout mice showed that CCR2-dependent monocytes were specifically required for the DDA/TDB-mediated induction of Th1/Th17 responses.

After immunization of *Ccr2^−/−^* mice the number of monocytes in the dLN was, as expected, severely reduced (**Figure 4D**). However, this lack of monocytes did not prevent the robust increase in total dLN cell numbers (**Figure 4D**), APC, or lymphocytes (**Supplementary Figure S5**). The number of adjuvant-containing total cells, including DC, in the dLN was also not affected in *Ccr2^−/−^* mice, suggesting that monocytes are not limiting for transfer of the antigen/adjuvant-containing liposomes to the dLN APC (**Figure 4E**). Together, although induction of Th1/Th17 immunity required signaling *via* Mincle and MyD88, and CCR2-dependent monocytes, genetic abrogation of these players did not impair transport of adjuvant to dLN DC.

## Discussion

The new generation synthetic glycolipid adjuvant DDA/TDB induces robust and persistent Th1/Th17 responses in animal models (9, 10, 14, 37) and promotes long-lived T-cell responses to the MTB fusion protein H1 in humans (5). In this manuscript, we have investigated the cellular recruitment to the injection site, the signaling mechanisms required, and the functional role of the most abundantly recruited cell types. Neutrophils and monocytes formed the major component of the cellular infiltrate and were recruited in a partially Mincle- and MyD88-dependent manner. The major finding of this study is the identification of a pivotal function of CCR2^+^ monocytes in the promotion of Th1/Th17-biased antigen-specific immunity by DDA/TDB. This requirement for monocytes was selective for DDA/TDB, as the Th1-inducing effect of the TLR9 ligand CpG ODN was not affected in *Ccr2^−/−^* mice. Neutrophils, although being the most abundantly recruited cell type after DDA/TDB injection, were dispensable for adjuvanticity, as shown by antibody-mediated depletion and confirmed in neutrophil-deficient *Mcl-1* conditional knockout mice. Thus, our results show a dichotomy in the functional roles of monocytes and neutrophils, with implications for future adjuvant optimization and investigations of cellular and molecular mechanisms.

Neutrophils can contribute to the development of adaptive immune responses by release of chemokines and cytokines attracting monocytes and DC and by the transport of antigen or whole microorganisms to lymphoid tissues (38, 39). However, strong neutrophil infiltration can also be associated with more severe side effects after vaccination, ranging from pain at the injection site to severe tissue damage (40). Therefore, our finding that a lack of neutrophils did not hinder the adjuvant effect of DDA/TDB implies the possibility of reducing unwanted inflammation due to granulocyte infiltration by developing and selecting adjuvants with a selective targeting of monocytes. On the other hand, the tendency for stronger Th1/Th17 induction observed here in mice depleted of neutrophils with 1A8 antibody (**Figure 3**) may indicate a negative regulatory effect of neutrophils, consistent with triggering of IL-10 release by the cord factor analog TDB (41) or the previously described chemokine binding and degrading function of neutrophil extracellular traps (42).

CCR2-dependent monocyte recruitment plays an essential role in the control of several bacterial and fungal infections by inducing protective immune responses (43–49). In addition, *Ccr2^−/−^* mice show diminished IFNγ responses upon immunization with Complete Freund’s Adjuvant (43), whose activity is partially due to the TDM of the killed mycobacteria and, similar to TDB, mediated by Mincle-Card9- and IL1R-MyD88-dependent signaling (15). In contrast, soluble CpG ODN-induced generation of Th1 cells was not affected by CCR2 deficiency (**Figure 4**). This differential requirement is consistent with a crucial role for monocytes in promoting the response to particulate vaccines in the dLN, where they may present antigen to T cells directly, transfer the antigen to DC, or create the appropriate cytokine milieu through release of soluble mediators.

Whether monocytes can directly prime naive T-cell responses is debated. Following immunization with aluminum hydroxide as adjuvant, monocytes recruited to the SOI take up antigen and differentiate to monocyte-derived DC (moDC) during their migration to the dLN (29). During infection with *Leishmania major*, moDC controls the induction of protective Th1 responses (50). Recruited monocytes differentiate into moDC and migrate to the dLN, and only these moDCs capture and present *L. major*-derived antigens. On the other hand, evidence against direct priming capabilities of moDC has been reported using a yeast vaccine strain expressing a model antigen (51). Despite association of yeast and moDC, peptide:MHCII complexes could not be detected on these cells. Instead, the material had to be transferred to LN-resident DC or migratory skin-derived DC, which were essential for priming of naive T cells (51). During infection with *M. tuberculosis*, inflammatory monocytes were necessary for CD4^+^ T-cell priming because they transported bacteria to the dLN. However, by transferring MHCII-deficient monocytes into monocyte-depleted hosts, it was shown that only conventional DC (cDC) were able to induce antigen-specific CD4^+^ T-cell proliferation (52). Recently, Bosteels et al. described that cDC2 in inflammatory conditions acquire a phenotype sharing characteristics with moDC (e.g., expression of CD64) and termed these cells inf-cDC2 (53). Mixed bone marrow chimeras showed that CCR2 was required for the presence of inf-cDC2 in the lungs after respiratory virus infection (53) and in the draining lymph nodes after immunization with the adjuvant AS01 (54).

In our experimental system, flow cytometry revealed no differences in cellular composition of the dLN of *Ccr2^−/−^* mice (except for the monocytes), and the numbers of adjuvant-positive cells were comparable. Thus, DDA/TDB adjuvant is targeted equally well to APC in the dLN in the absence of monocytes. Therefore, the essential function of the monocytes recruited to the SOI and the dLN may be to provide cytokines and mediators acting as signal 3 to direct the T-cell response in the dLN towards Th1/Th17. Candidates to be tested in future experiments include IL-1, shown by us and others to direct Th1/Th17 induction by DDA/TDB (14, 15), and IL-6, which is produced by human monocytes and important for Th17 differentiation (55).

The rapid and striking increase in cell numbers in the dLN following DDA/TDB administration is likely due to trapping of cells rather than to proliferation. A shutdown phase, leading to a dramatic reduction in efferent cell output, has been described (56) and shown to be mediated by TNFα and IL-6 (57). We detected IL-6 release already 2 h p.i. in peritoneal lavage fluids. In addition, IFNα/β-mediated upregulation of CD69 on T cells contributes to their retention within lymphoid organs (58); however, we have not addressed induction of type I interferon upon immunization here. Trapping of cells in the dLN maximizes the number of naive antigen-specific T cells available for encounter with antigen-loaded APC. Cell trapping was a MyD88-dependent effect predominantly induced by DDA during the first 24 h, whereas a further Mincle- and MyD88-dependent increase in cell numbers at d3 p.i. was only observed when TDB is incorporated into the liposomes. Remarkably, CCR2 deficiency had no effect on lymph node cellularity following a single immunization with DDA/TDB, indicating that the factors mediating lymph node shutdown are produced independently of monocytes and are insufficient to direct Th1/Th17 responses.

In conclusion, our study provides evidence that Th17 immunity induced by a Mincle-targeting adjuvant depends on the recruitment of CCR2^+^ inflammatory monocytes. In contrast, the abundantly and rapidly recruited neutrophils were completely dispensable for the adjuvant effect. How exactly the monocytes promote Th1/Th17 generation remains to be elucidated. Since the Mincle- and MyD88-dependent increase in lymph node cellularity and the appearance of vaccine-containing APC in the draining lymph node were not compromised in CCR2-deficient mice, the contribution of monocytes may consist in the activation of APC or provision of signal 3 to T cells.

## Materials and Methods

### Ethics Statement

All procedures were discussed with and approved by the animal protection committee of the regional Bavarian government (Regierung von Mittelfranken animal protocol numbers 54-2532.1-12/09 and -39/13) according to the German animal protection law and the Institutional Animal Care and Use Committee of St. Jude Children’s Research Hospital (#267).

### Mice and Immunization

*Myd88* ^−/−^ mice were used with permission of Dr. S. Akira (34), *Mincle* ^−/−^ mice have been described (59) and were used with permission by the Consortium for Functional Glycomics. *Ccr2* ^−/−^ mice were obtained from Jackson Laboratories. C57BL/6, *Mincle* ^−/−^, *Myd88* ^−/−^, and *Ccr2* ^−/−^ mice were bred at the animal facility of the Medical Faculty in Erlangen. *Mcl-1^f^* ^lox/flox^;LysM-Cre conditional knockout mice were bred and immunized at St. Jude Children’s Research Hospital. For some experiments, C57BL/6 mice were purchased from Charles River. Liposomes and recombinant H1 were provided by the Statens Serum Institut. Adjuvant formulations were prepared as described (10). Mice were injected with 100 µl i.p. or 2 × 50 µl liposomes or 10 nmol CpG 1826 s.c. into the hind footpads. Two micrograms of H1 was only added if antigen-specific re-stimulation was performed. In some experiments, 7-nitrobenzo-2-oxa-1,3-diazole (NBD)-fluorescently labeled liposomes were injected in order to visualize the adjuvant.

### Generation and Stimulation of Macrophages and DC

Bone marrow cells were cultured on Petri dishes for 8 days in Roswell Park Memorial Institute (RPMI) containing 10% X63 (BM-DC) or for 6 days in complete Dulbecco’s modified Eagle’s medium (cDMEM) containing 10% L929 (BMM) cell-conditioned medium. Stimulation is indicated with plate-coated TDB, TDM (10), or LPS. TNFα or G-CSF release was determined by ELISA (R&D Systems).

### Antigen-Specific Re-Stimulation

Lymph nodes were meshed through a 100-µm nylon sieve and 5 × 10^5^ cells re-stimulated with 10 µg/ml of H1 protein for 96 h. Supernatants were analyzed for IFNγ and IL-17 production by ELISA (R&D Systems, Minneapolis, USA). Background (unstimulated cells) was subtracted.

### Cell Isolation and Flow Cytometry

For peritoneal lavage, 2 ml of cold phosphate-buffered saline (PBS)/0.2% bovine serum albumin (BSA) was injected i.p., and the cell suspension was carefully aspirated and counted to determine the number of cells in the peritoneum. Cells were centrifuged, supernatant was collected for the determination of chemokines by ELISA (R&D Systems), and cells were stained for fluorescence-activated cell sorting (FACS) analysis. Cells from inflamed feet were isolated using a GentleMACS dissociator (Miltenyi, Bergisch-Gladbach, Germany) according to manufacturers’ protocol for splenocytes and counted. Cells were stained with fluorochrome-conjugated antibodies against B220, CD3, CD4, CD8, CD11b, CD11c, F4/80, γδTCR, Gr-1, Ly6C, Ly6G, MHC class II, NK1.1, or PDCA (BioLegend or eBioscience, San Diego, CA, USA). Examples of gating strategies are depicted in **Supplementary Figure S1A**. Non-specific binding of antibodies was blocked by 15 min incubation of cells with Fc-receptor blocking antibody. Data were recorded on a FACSCanto™ II and analyzed with FACSDiva™ 6.1 (BD Biosciences, Heidelberg, Germany). Results are presented in absolute numbers of cells calculated using the cell counts after isolation and the percentage of each respective gated cell subset.

### Cell Depletion

For neutrophil depletion, 100 µg of Ly6G-specific antibody 1A8 (Bio X Cell, Lebanon, NH, USA) was injected i.p. in 48-h interval starting 24 h prior to vaccination. Monocytes were depleted using 10 µg of CCR2-specific MC-21 antibody i.p. every 24 h starting 1 h prior to vaccination (33); 10 µg of the isotype control LEAF^TM^ purified rat IgG2bk (BioLegend) was injected i.p. daily.

## Data Availability Statement

The original contributions presented in the study are included in the article/**Supplementary Material**. Further inquiries can be directed to the corresponding authors.

## Ethics Statement

The animal study was reviewed and approved by Animal Protection Committee Regierung von Mittelfranken.

## Author Contributions

CD performed most experiments. CD and RL designed experiments and analyzed data. PM and LH performed experiments. PM, CL, LH, DC, PA, DD, and MM provided reagents and contributed to writing of the manuscript. CD and RL wrote the manuscript. All authors contributed to the article and approved the submitted version.

## Conflict of Interest

PA and DC are co-inventors of patents relating to cationic liposomes. All rights have been signed to Statens Serum Institute.

The remaining authors declare that the research was conducted in the absence of any commercial or financial relationships that could be construed as a potential conflict of interest.

## Publisher’s Note

All claims expressed in this article are solely those of the authors and do not necessarily represent those of their affiliated organizations, or those of the publisher, the editors and the reviewers. Any product that may be evaluated in this article, or claim that may be made by its manufacturer, is not guaranteed or endorsed by the publisher.

## Supplementary Material

Supplementary Figure 1 Gating strategies for flow cytometry. **(A)** Boolean gating strategies used: Neutrophils G1&P1&P2 (CD11b^+^MHCII^-^Ly6C^+^Ly6G^+^, blue) and monocytes G1&P3&P4 (CD11b^+^Ly6C^+^Ly6G^-^, red); macrophages: G1&P5 (F4/80^++^CD11b^++^); DC: G1&P6&P7 (F4/80^-^CD11c^+^MHCII^+^); B cells: G2&P8 (B220^+^ cells in leukocyte gate); CD4 T cells: G2&P9 (CD4^+^ cells in leukocyte gate); CD8 T cells: G2&P10 (CD8^+^ cells in leukocyte gate); NK cells: G2&P11 (NK1.1^+^ cells in leukocyte gate); γδTCR^+^ cells: G2&P12 (γδTCR^+^ cells in leukocyte gate); eosinophils: G3&P13 (Ly6C^low^ expressing SSC^++^ cells) and pDC: G1&P15&P14 (CD11b^-^PDCA^+^CD11c^+^ cells). **(B)** Recruitment kinetics for cell populations analyzed from peritoneal lavage, DDA: open circles, DDA/TDB: closed spheres. Pooled data from 8 independent experiments, n= 5-9 mice per group/time point, cell numbers shown as mean+SD. 2-way ANOVA and Bonferroni correction; p<0.05=*, p<0.01=**, p<0.001=***. **(C)** Cell recruitment footpad; injection of NBD-DDA/TDB. Pooled data from 7 independent experiments with total n=5-9 mice per group and time point. Data presented as mean+SD. Significance tested by 1-way ANOVA and Dunnett’s posttest (day 0 as control group).

Supplementary Figure 2 APC numbers and acquisition of adjuvant in the dLN. **(A)** Numbers of APC in popliteal lymph nodes 1d and 3d after s.c. footpad immunization with NBD-DDA/TDB. **(B)** Numbers of adjuvant-positive APC; popliteal lymph nodes 1d and 3d p.i. Pooled data from 5 independent experiments.

Supplementary Figure 3 Depletion efficacy of MC-21 (monocytes) or 1A8 (neutrophils). **(A)** Gating strategies for monocytes: P1&P3 (CD11b^+^Ly6C^+^Gr-1^int^) and neutrophils: P1&P2 (CD11b^+^Ly6C^+^Gr-1^high^) and dot plot examples. **(B)** Percentages in blood, pooled popliteal and inguinal lymph nodes or cells isolated from the footpad 7d p.i.; 5 mice per group. One of two experiments shown. **(C)** % monocytes and % neutrophils **(D)** in blood, popliteal lymph nodes or cells isolated from the footpad 24h p.i.; 3 mice per group, one experiment. Data presented as mean+SD; 1-way ANOVA and Dunnett’s post test (isotype as control group) p<0.05=*, p<0.01=**, p<0.001=***.

Supplementary Figure 4 CCR2^−/−^ do not have a general defect in Th1/Th17 response generation. **(A)** IFNγ and IL-17 secretion after 48h polyclonal αCD3/αCD28 stimulation (5µg/ml) of 5x10^5^ splenocytes. Mean+SD; 2 mice per group, 1 of 2 representative. **(B)** TNF-α (bone marrow-derived macrophages) and G-CSF (bone marrow-derived DC) secretion after 48h stimulation with 100ng/ml LPS, 1.25µg/ml or 2.5µg/ml TDB or 1.25µg/ml TDM. Mean+SD from triplicates; 2.5x10^5^ cells; 1 of 2 representative. (A, B) C57BL/6 (white bars), *Ccr2* ^−/−^ (black bars). **(C)** Footpad swelling and cell counts from feet 7d post footpad immunization with DDA/TDB/H1 or CpG/H1.

Supplementary Figure 5 Cellular composition of the dLN is not altered in the absence of CCR2. Numbers of cells isolated from popliteal lymph nodes 1d and 3d after footpad immunization with NBD-DDA/TDB. Pooled data from 2 independent experiments with a total of 5-6 mice per group/time point. Significance tested by t-test.
