# Supplementary material for: Monocytes Elicit a Neutrophil-Independent Th1/Th17 Response Upon Immunization With a Mincle-Dependent Glycolipid Adjuvant
Source: Front Immunol. 2022 May 2;13:880474. doi: 10.3389/fimmu.2022.880474 (PMC9108773; doi:10.3389/fimmu.2022.880474)

Supplementary Fig. S1

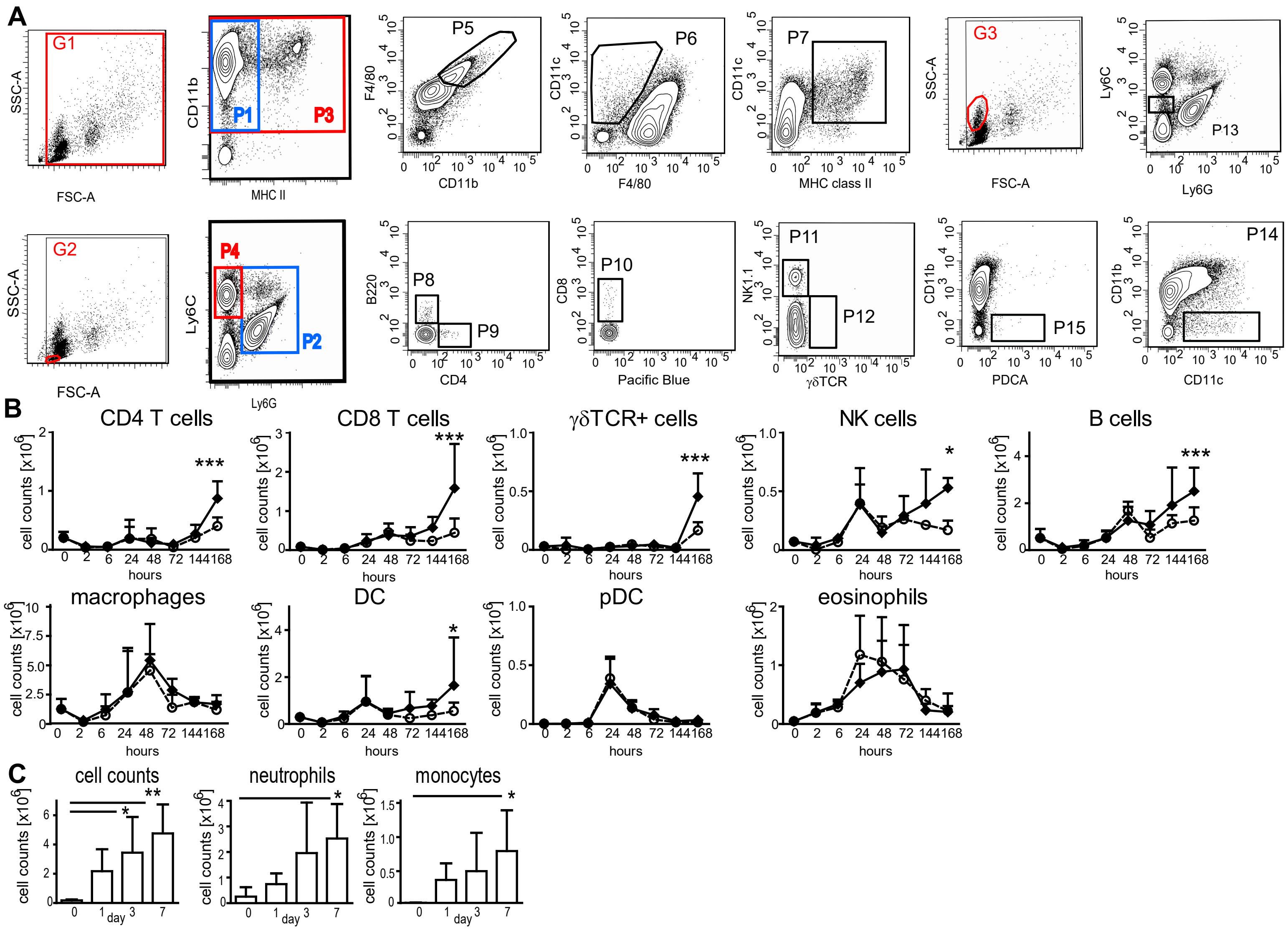

Supplementary Fig. S2

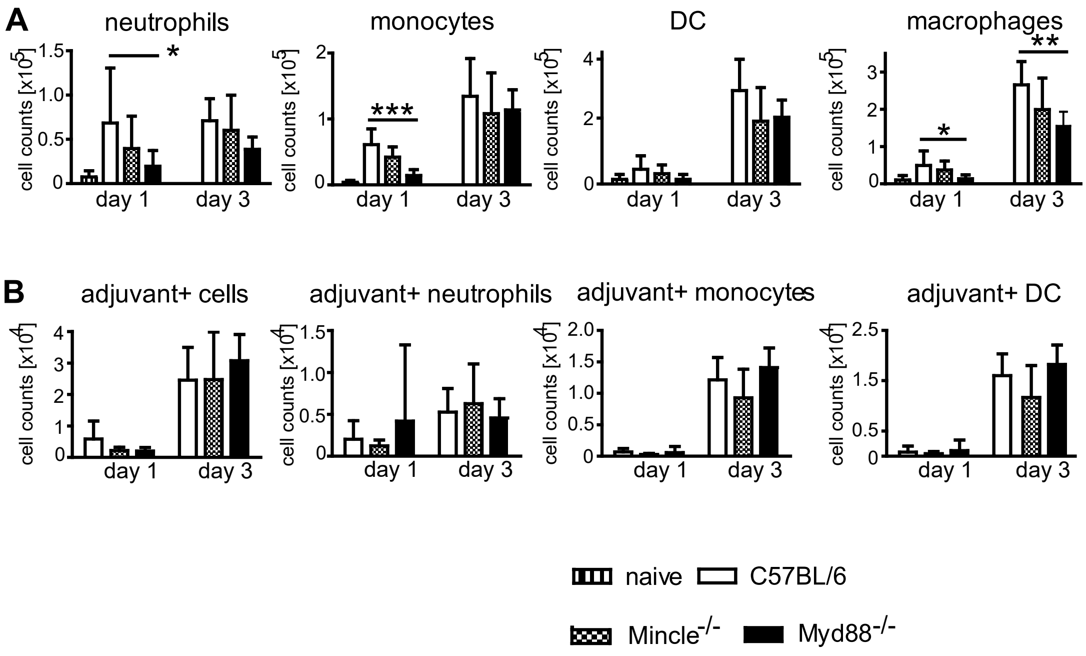

Supplementary Fig. S3

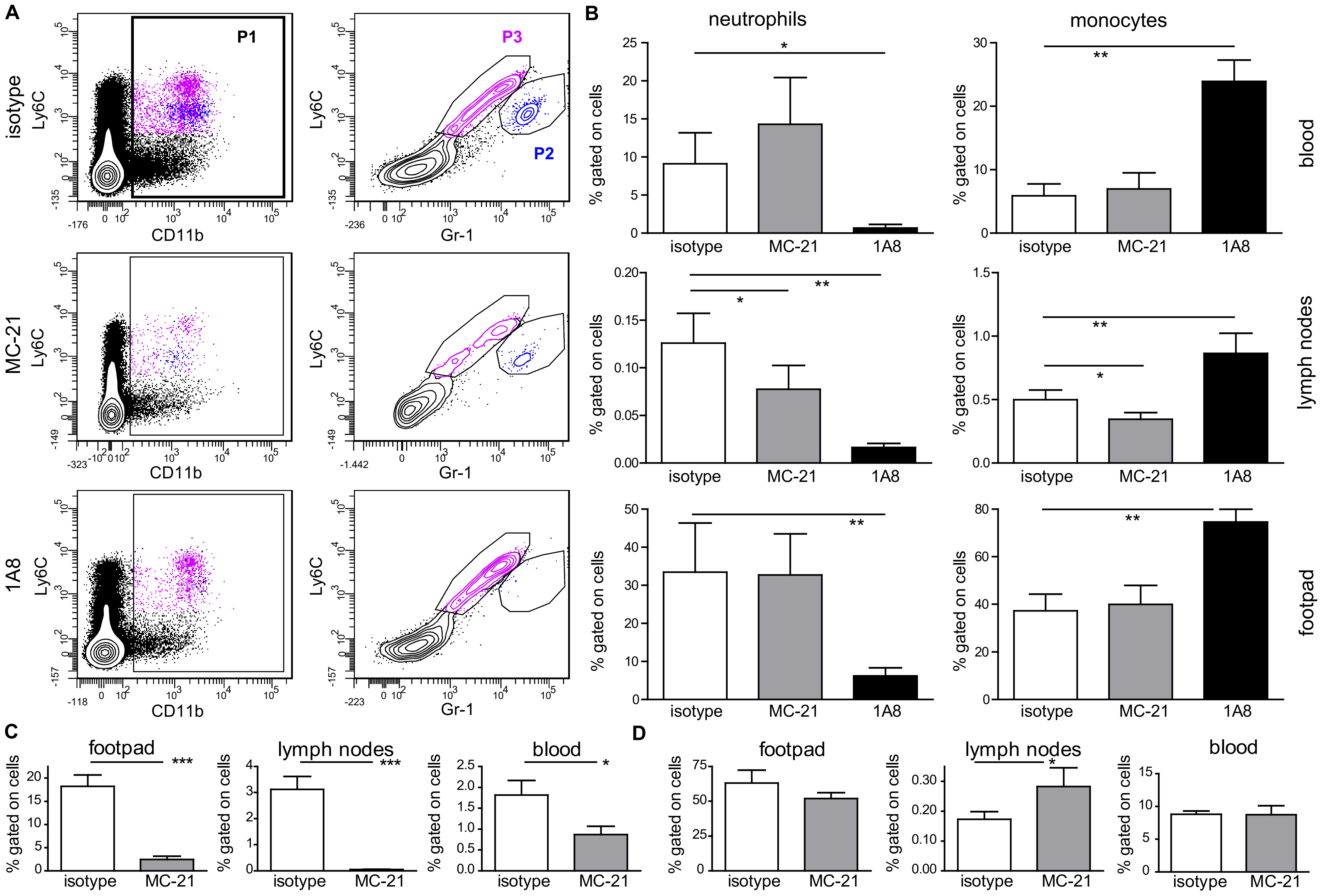

Supplementary Fig. S4

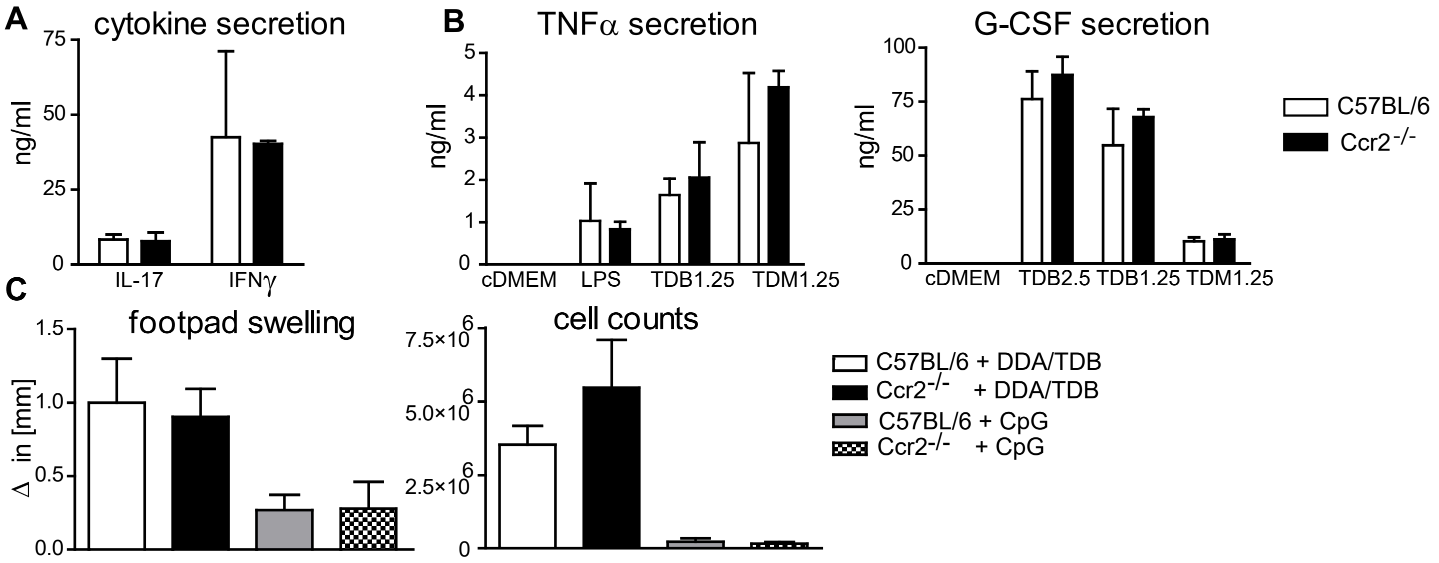

Supplementary Fig. S5

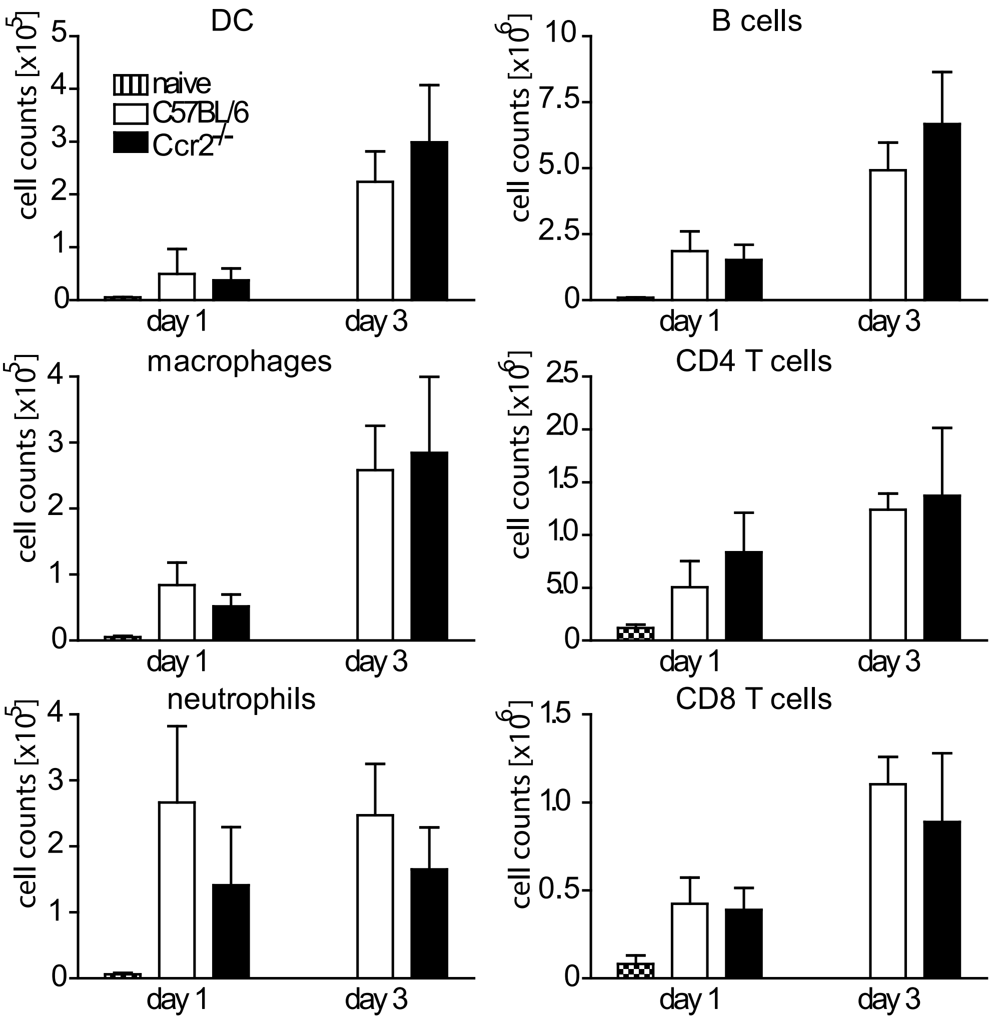

Supplement: Supplementary Figure 1 — Gating strategies for flow cytometry. (A) Boolean gating strategies used: Neutrophils G1&P1&P2 (CD11b+MHCII-Ly6C+Ly6G+, blue) and monocytes G1&P3&P4 (CD11b+Ly6C+Ly6G-, red); macrophages: G1&P5 (F4/80++CD11b++); DC: G1&P6&P7 (F4/80-CD11c+MHCII+); B cells: G2&P8 (B220+ cells in leukocyte gate); CD4 T cells: G2&P9 (CD4+ cells in leukocyte gate); CD8 T cells: G2&P10 (CD8+ cells in leukocyte gate); NK cells: G2&P11 (NK1.1+ cells in leukocyte gate); γδTCR+ cells: G2&P12 (γδTCR+ cells in leukocyte gate); eosinophils: G3&P13 (Ly6Clow expressing SSC++ cells) and pDC: G1&P15&P14 (CD11b-PDCA+CD11c+ cells). (B) Recruitment kinetics for cell populations analyzed from peritoneal lavage, DDA: open circles, DDA/TDB: closed spheres. Pooled data from 8 independent experiments, n= 5-9 mice per group/time point, cell numbers shown as mean+SD. 2-way ANOVA and Bonferroni correction; p<0.05=*, p<0.01=**, p<0.001=***. (C) Cell recruitment footpad; injection of NBD-DDA/TDB. Pooled data from 7 independent experiments with total n=5-9 mice per group and time point. Data presented as mean+SD. Significance tested by 1-way ANOVA and Dunnett’s posttest (day 0 as control group). [file DataSheet_1.pdf]
